# Anti‐tumor effects of an antagonistic mAb against the ASCT2 amino acid transporter on *KRAS*‐mutated human colorectal cancer cells

**DOI:** 10.1002/cam4.2689

**Published:** 2019-11-10

**Authors:** Yuta Hara, Yushi Minami, Soshi Yoshimoto, Natsumi Hayashi, Akitaka Yamasaki, Shiho Ueda, Kazue Masuko, Takashi Masuko

**Affiliations:** ^1^ Cell Biology Laboratory School of Pharmacy Kindai University Higashi‐Osaka Osaka Japan

**Keywords:** ASCT2, CRC, glutamine, KRAS, mAb

## Abstract

*KRAS* mutations are detected in numerous human cancers, but there are few effective drugs for *KRAS*‐mutated cancers. Transporters for amino acids and glucose are highly expressed on cancer cells, possibly to maintain rapid cell growth and metabolism. Alanine‐serine‐cysteine transporter 2 (ASCT2) is a primary transporter for glutamine in cancer cells. In this study, we developed a novel monoclonal antibody (mAb) recognizing the extracellular domain of human ASCT2, and investigated whether ASCT2 can be a therapeutic target for *KRAS*‐mutated cancers. Rats were immunized with RH7777 rat hepatoma cells expressing human ASCT2 fused to green fluorescent protein (GFP). Splenocytes from the immunized rats were fused with P3X63Ag8.653 mouse myeloma cells, and selected and cloned hybridoma cells secreting Ab3‐8 mAb were established. This mAb reacted with RH7777 transfectants expressing ASCT2‐GFP proteins in a GFP intensity‐dependent manner. Ab3‐8 reacted with various human cancer cells, but not with non‐cancer breast epithelial cells or *ASCT2*‐knocked out HEK293 and SW1116 cells. In SW1116 and HCT116 human colon cancer cells with *KRAS* mutations, treatment with Ab3‐8 reduced intracellular glutamine transport, phosphorylation of AKT and ERK, and inhibited in vivo tumor growth of these cells in athymic mice. Inhibition of in vivo tumor growth by Ab3‐8 was not observed in HT29 colon and HeLa uterus cancer cells with wild‐type *KRAS*. These results suggest that ASCT2 is an excellent therapeutic target for *KRAS*‐mutated cancers.

## Graphical abstract

ASCT2 is a major glutamine transporter in various cancer cells, and higher expression of ASCT2 is correlated with poor prognosis of cancer patients. This study strongly suggests that ASCT2 is an excellent target molecule for the treatment of malignancies, especially *KRAS*‐mutated cancers.

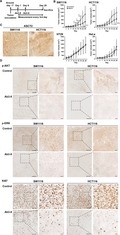

## 1 INTRODUCTION

Colon or rectal (colorectal) cancer (CRC) is one of the leading causes of cancer‐related mortality,^1^ and the worldwide incidence and death rates have increased, especially in developing countries.^2^ Multiple gene mutations have been reported in patients with CRC, especially, *KRAS* mutations are detected in approximately 40% of patients,^3^ whereas NRAS and BRAF mutations are less common.^4^, ^5^ In addition to surgical resection and radiotherapy, chemotherapy and molecular target therapy are used in CRC. Cetuximab and panitumumab, therapeutic monoclonal antibodies (mAb) against epidermal growth factor receptor (HER1/EGFR), are frequently used, but are less effective in a subset of cancers with *KRAS* mutations.^6^ In clinical trials in patients with *KRAS*‐mutated CRC, multiple combination therapies such as AKT inhibitor‐MEK inhibitor,^7^ MEK inhibitor‐FOLFIRI,^8^ and cetuximab‐FOLFOX^9^ have shown disappointing results. Similar to strategies to suppress KRAS signaling pathway and function, developing attempts at direct targeting KRAS is anticipated to be long and difficult way.^10^ As mentioned above, there are no effective therapies for *KRAS*‐mutated CRC, and furthermore, the exact molecular pathology of CRC with *KRAS* mutations is still unsolved.

Glutamine is considered as a conditional essential amino acid for proliferation and survival of cancer cells.^11^, ^12^, ^13^ In particular, *KRAS*‐mutated cancer cells are reported to be prone to increase the demand of glutamine.^14^ Indeed, several studies demonstrated the effects of oncogenic *KRAS* on glutamine metabolism. Utilization of glutamine for anabolic synthesis and the expression of genes associated with glutamine metabolism are upregulated in NIH3T3 cells expressing *KRAS*‐mutated proteins and in *KRAS*‐mutated human breast cancers.^15^ In pancreatic cancers, oncogenic *KRAS* plays an important role in the reprogramming of glutamine metabolism.^16^ Furthermore, glutamine catabolism in the TCA cycle is necessary for KRAS‐induced anchorage‐independent growth.^17^ Taken together, glutamine plays an essential role in the growth of *KRAS*‐mutated cells.

ASCT2 (*SLC1A5*) is a multi‐pass transmembrane protein that transports neutral amino acids. In many cancer cells, ASCT2 is highly expressed and regarded as a primary transporter for glutamine.^18^ Several studies have reported that treatment with ASCT2 inhibitor or knockdown of *ASCT2* causes marked decreases in intracellular glutamine concentration and cell viability in various human cancer cells.^19^, ^20^, ^21^, ^22^, ^23^, ^24^ In human tumor tissues harboring *KRAS* mutation, ASCT2 proteins were highly expressed as compared with *KRAS* wild‐type tumors.^25^ Although the relationship between *KRAS* mutations and ASCT2 remains unclear, inhibition of ASCT2 function may be a promising method to treat *KRAS*‐mutated cancers considering the importance of glutamine in cells with *KRAS* mutation. In this context, we developed specific mAb recognizing the extracellular domain of human ASCT2 and examined the effects of mAb on in vitro and in vivo growth of *KRAS*‐mutated human colon cancer cells.

## 2 MATERIALS AND METHODS

### 2.1 Cell culture

Following cell lines were purchased from American Type Cell Collection (ATCC): HT29 (#HTB‐38), SW1116 (#CCL‐233), HCT15 (#CCL‐225), DLD‐1 (#CCL‐221), HCT116 (#CCL‐247), BT20 (#HTB‐19), T47D (#HTB‐133), AU565 (#CRL‐2351), MDA‐436 (#HTB‐130), H2170 (#CRL‐5928), H1975 (#CRL‐5908), HeLa (#CRM‐CCL‐2), T24 (#HTB‐4), HME1 (#CRL‐4010), and MCF10A (#CRL‐10317). ABC‐1 (#JCRB0815) was purchased from Japanese Collection of Research Bioresources (JCRB). Tagawa was provided by Dr Ohuchi N, Second Department of Surgery, School of Medicine, Tohoku University. Cells were cultured in equal volumes of RPMI‐1640 medium and Dulbecco's modified Eagle's medium (RD medium; #05918 and #05919, Nissui Pharmaceutical Co., Ltd) supplemented with 7% fetal bovine serum (FBS; #10270‐106, Thermo Fisher Scientific Inc) in humidified CO_2_ incubators. RH7777 rat hepatoma (#CRL‐1601; provided by Dr Chiba, Tanabe Mitsubishi Pharm) and HEK293F human embryonic kidney cells (#R79007, Invitrogen) expressing human ASCT2 fused to green fluorescent protein (GFP) (ASCT2‐GFP) were cultured in RD medium with 7% FBS and 0.4 mg/mL of G418 (#09380‐44, Nacalai Tesque).

### 2.2 Establishment of *ASCT2*‐knocked out (KO) cells

pX330‐U6‐Chimeric_BB‐CBh‐hSpCas9 (#42230)^26^ and pCAG‐EGxxFP (#50716)^27^ plasmids were purchased from Addgene. For CRISPR/Cas9‐based *ASCT2* gene disruption, guide (g) RNA sequences (5′‐GCGGAGCCCACCGCCAACGG‐3′) corresponding to *ASCT2* gene (43 bp‐62 bp from the initiation ATG site) were designed using CRISPR direct (https://crispr.dbcls.jp/). The efficiency of KO by pX330 plasmids expressing codon‐optimized SpCas9 and chimeric gRNA was confirmed by double‐strand break‐mediated enhanced GFP reconstitution with co‐transfection of pX330 and pCAG‐EGxxFP plasmids into HEK293F cells. Cells were seeded into 35‐mm dishes in 1 mL of RD medium with 7% FBS, grown to 80% confluency, and plasmid DNA (5 μg) was introduced into these cells using Xfect transfection reagent (#631317, Takara Bio Inc).

### 2.3 Animals

Six‐week‐old female F344/N rats and 6‐week‐old male KSN athymic (nude) mice were purchased from SLC Inc (Hamamatsu, Japan). They were housed in specific pathogen‐free conditions, kept individually in cages under a standard light/dark cycle (12‐hr light cycle starting at 7:00) at a constant temperature of 23 ± 1°C, and had ad libitum access to food and water. Animal experiments in this study were approved by the Committee for the Care and Use of Laboratory Animals at Kindai University, and performed following the institutional guidelines and the United States National Institutes of Health Guide for the Care and Use of Laboratory Animals.

### 2.4 Rat mAb against human ASCT2

Production of the anti‐human ASCT2 mAb was performed according to previous reports.^28^, ^29^ In brief, RH7777 cells expressing human ASCT2‐GFP were injected three times into F344/N rats every 2 weeks. Three days after the final immunization, cell fusion was carried out by mixing the splenocytes of immunized rats with P3X63Ag8.653 mouse myeloma cells (#CRL‐1580, ATCC) using 50% polyethylene glycol (#10783641001, Roche). Hybridomas were chosen for their binding capacity of antibodies in culture supernatant to transfectants expressing ASCT2. Selected and cloned hybridoma cells were injected into athymic mice pretreated with 2,6,10,14‐tetramethylpentadecane (Pristane; #161‐27483, Wako). Ascites was harvested and purified using Protein G sepharose (#17061801, GE Healthcare). The isotype of mAb was determined with the Rapid Monoclonal Antibody Isotyping Kit (#ISO‐M6‐20, Antagen Pharmaceuticals, Inc).

### 2.5 Flow cytometry (FCM)

FCM was performed as previously described.^28^, ^29^ For the screening of hybridomas, RH7777 or HEK293 (1 × 10^6^ cells) expressing ASCT2‐GFP were reacted with undiluted hybridoma culture supernatants, followed by the incubation with PE‐conjugated donkey anti‐rat IgG (1:300; #712‐116‐153, Jackson ImmunoResearch Inc). For measurement of ASCT2 proteins on the cell surface, cells (1 × 10^6^) were stained with 10 μg/mL of Ab3‐8, followed by incubation with PE‐conjugated above secondary antibody. Between the incubation steps, cells were washed with Dulbecco's phosphate‐buffered saline (PBS) containing 0.2% bovine serum albumin (#01281‐84, BSA; Nacalai Tesque). For two‐color immunostaining, cells (1 × 10^6^) were fixed with 4% paraformaldehyde (PFA; #162‐16065, Wako) in PBS for 15 minutes, and incubated in 90% methanol at 4°C for 30 minutes for permeabilization. The cells were reacted with a combination of Ab3‐8 (10 μg/mL) and anti‐ASCT2 rabbit mAb (1:200; #8057, Cell Signaling Technology, Inc) at room temperature for 1 hour, and reacted with Alexa Fluor 488‐labeled anti‐rat IgG (1:200; #712‐545‐153) and Alexa Fluor 647‐labeled anti‐rabbit IgG (1:200; #711‐605‐152) (Jackson ImmunoResearch) at 4°C for 45 minutes. The fluorescence intensity of each cell was measured by a flow cytometer (BD LSR Fortessa; Becton‐Dickinson) and analyzed using FlowJo software (TreeStar). Cells stained with only fluorochrome‐conjugated secondary antibodies, but not Ab3‐8 or anti‐ASCT2 rabbit mAb, were used as the control.

### 2.6 Immunoprecipitation

Immunoprecipitation was performed according to our previous report.^28^ Briefly, cells were lysed in the buffer containing 50 mmol/L Tris‐HCl (pH 7.4), 150 mmol/L NaCl, 1% Nonidet P‐40 (#18551‐95, Nacalai Tesque), and protease inhibitor cocktail (#04080‐24, Nacalai Tesque). Cell lysates were incubated with Ab3‐8 (20 μg) at 4°C overnight, and Protein G sepharose was added. The precipitated proteins were separated by SDS‐PAGE. The following procedure was described in “Western blotting” in the materials and methods section with anti‐ASCT2 rabbit mAb.

### 2.7 Immunocytochemistry

Cells (1 × 10^5^) were plated on Geltrex (#A1413202, Thermo Fisher Scientific)‐coated eight‐well chamber slides (#192‐008, WATSON Bio Lab). The cells were fixed with 4% PFA‐PBS for 15 minutes and coated with Block Ace (#UKB40, Dainippon Pharmaceutical Co.) containing 0.3% Triton X‐100 (#12969‐25, Nacalai Tesque) for 1 hour at room temperature. Subsequently, the cells were reacted with Ab3‐8 (10 μg/mL) and anti‐ASCT2 rabbit mAb (1:200) overnight at 4°C. The cells were incubated with Alexa Fluor 488‐labeled anti‐rat IgG (1:200) and Alexa Fluor 647‐labeled anti‐rabbit IgG (1:200) for 2 hours at room temperature. The confocal fluorescence images were obtained by confocal laser fluorescence microscopy using FV10C‐O (Olympus, Tokyo, Japan) with a 60 × oil immersion lens. We observed at least 30 cells in each cell line.

### 2.8 Internalization of ASCT2 proteins

Microscopy study of direct internalization: HEK293 and RH7777 cells overexpressing human ASCT2‐GFP were used. Cells were incubated with Ab3‐8 (10 μg/mL) for 1 hour at 37°C. Fluorescence images were obtained using a BioZero microscope (Keyence) with a 20 × Plan Fluor objective lens. We observed at least 20 cells in each cell line. Quantification analysis using FCM: Cells (2 × 10^6^) were reacted with Ab3‐8 (10 μg/mL) at 4°C for 30 minutes. After that, the cells were divided into two groups; one was incubated at 4°C, and the other did at 37°C for 1 hour. Cells were reacted with PE‐labeled anti‐rat IgG at 4°C for 1 hour, followed by FCM analysis.

### 2.9 Glutamine‐dependent cell proliferation assay

Cells (2 × 10^3^) were seeded onto each well of 96‐well plates in serum‐ and glutamine‐free DMEM medium (#A1443001, Thermo Fisher Scientific) containing 7% dialyzed FBS, 1 mg/mL of D‐glucose, and 0.11 mg/mL of sodium pyruvate. The cells were cultured for 3 days in the absence or presence of 2 mmol/L glutamine, and were measured for cell proliferation every 24 hours. For the evaluation of cell proliferation, WST‐8‐based Cell Count Reagent SF (#07553‐44, Nacalai Tesque) was added to each well, and incubated for 3 hours at 37°C. After the incubation, the absorbance was read at 450 nm using a FilterMax F3 microplate reader (Molecular Devices, LLC). The assay was performed in duplicate.

### 2.10 Glutamine uptake

Cells (2 × 10^6^) were cultured in 35‐mm culture dishes overnight. After cells were incubated for 2 hours with Ab3‐8 (30 μg/mL) in PBS containing 1 g/L of D‐glucose and 0.11 mg/mL of sodium pyruvate, L‐glutamine (2 mmol/L) was added for 10 minutes. Concentrations of intracellular glutamine were measured in cell lysates using the glutamine/glutamate‐Glo Assay kit (#J8021, Promega Co.) according to the manufacturer's instructions. Luminescence was measured using a FilterMax F3 microplate reader. The data were normalized to total protein levels and this assay was performed in duplicate.

### 2.11 Western blotting

Cells (3 × 10^6^) were starved for 24 hours in serum‐ and glutamine‐free DMEM medium adding 1 g/L of D‐glucose and 0.11 mg/mL of sodium pyruvate in the presence of Ab3‐8. Fifteen min after the addition of glutamine, cells were lysed in lysis buffer containing 50 mmol/L Tris‐HCl (pH 7.4), 150 mmol/L NaCl, 1% Nonidet P‐40, 0.1% SDS, protease inhibitor cocktail, and phosphatase inhibitor cocktail (#07574‐61, Nacalai Tesque) for 15 minutes at 4°C. The cell lysates were centrifuged for 10 minutes at 15 000 × *g* at 4°C. Protein concentrations were measured using a BCA Protein Assay Kit (#T9300A, Takara Bio). Proteins (15 μg) were boiled for 5 minutes, and separated by SDS‐polyacrylamide gel electrophoresis (reduced; 8%). The samples were loaded in duplicate. The proteins were transferred electrophoretically to a hydrophobic PVDF membrane (#IPVH00010, pore size 0.45 μm, Merck Millipore). The blotted membranes were blocked for 1 hour at room temperature in 3% skim milk/Tris‐buffered saline (20 mmol/L Tris‐HCl, 137 mmol/L NaCl and 0.1% Tween 20, pH 7.6) and incubated overnight at 4°C with the following antibodies: phospho‐Akt (Ser473; #4060), phospho‐p44/42 MAPK (Erk1/2) (Thr202/Tyr204; #9101), Akt (#4691), p44/42 MAPK (Erk1/2) (#9102), and GAPDH (#2118). Membranes were incubated with horseradish peroxidase (HRP)‐conjugated anti‐rabbit IgG (1:5000; #711‐035‐152, Jackson ImmunoResearch) for 2 hours at room temperature. The immune complexes were visualized using Chemi‐Lumi One Super (#02230, Nacalai Tesque) and an ImageQuant RT ECL Imager (GE Healthcare). All primary antibodies were purchased from Cell Signaling and diluted 1:2000 (except for GAPDH 1:5000) in 3% skim milk/TBS‐T. The band densitometry was quantified using Image J Software (US National Institutes of Health).

### 2.12 In vivo tumor formation

The in vivo tumor study was performed as previously described.^28^, ^29^ Tumor‐bearing mice were prepared by subcutaneous inoculation of 5 × 10^6^ of human colon cancer cells or 1.4 × 10^6^ of HeLa cells in the left or right flank. The day on which the tumor was engrafted was defined as day 1. Tumor volumes were measured every third day using digital calipers, and were quantified using the formula: volume [mm^3^] = (length [mm]) × (width [mm])^2^ × 0.5. Ab3‐8 (100 μg/mouse) was administered intraperitoneally on days 1 and 9.

### 2.13 Immunohistochemistry

Immunohistochemistry was performed as previously described.^28^ In brief, serial 10‐μm‐thick sections were fixed with 4% PFA‐PBS for 10 minutes and blocked with Block Ace for 1 hour. The sections were incubated overnight with the following antibodies: phospho‐Akt (Ser473), phospho‐ERK (Thr202/Tyr204), and biotinylated Ki‐67 (#13‐5699, Thermo Fisher Scientific). They were reacted with biotinylated anti‐rabbit IgG (1:1000; #111‐065‐144, Jackson ImmunoResearch), except for anti‐Ki‐67 antibody‐treated sections, for 2 hours, followed by incubation with Elite ABC solution (#PK‐6100, Vector Laboratories) for 1 hour. After that, the sections were colored with 3,3′‐diaminobenzidine (DAB, #347‐00904, Dojin Chemicals, Kumamoto, Japan). Images were obtained using a BioZero microscope with a 4 × and 20 × Plan Apo objective lens.

### 2.14 Statistical analysis

Data are presented as mean ± standard deviation (SD). The significance of the differences was determined by the Mann‐Whitney U test using GraphPad Prism 7 for Windows (GraphPad Software). The criterion for significance was *P* < .05.

## 3 RESULTS

### 3.1 Production of a novel anti‐human ASCT2 mAb

**Figure 1:**
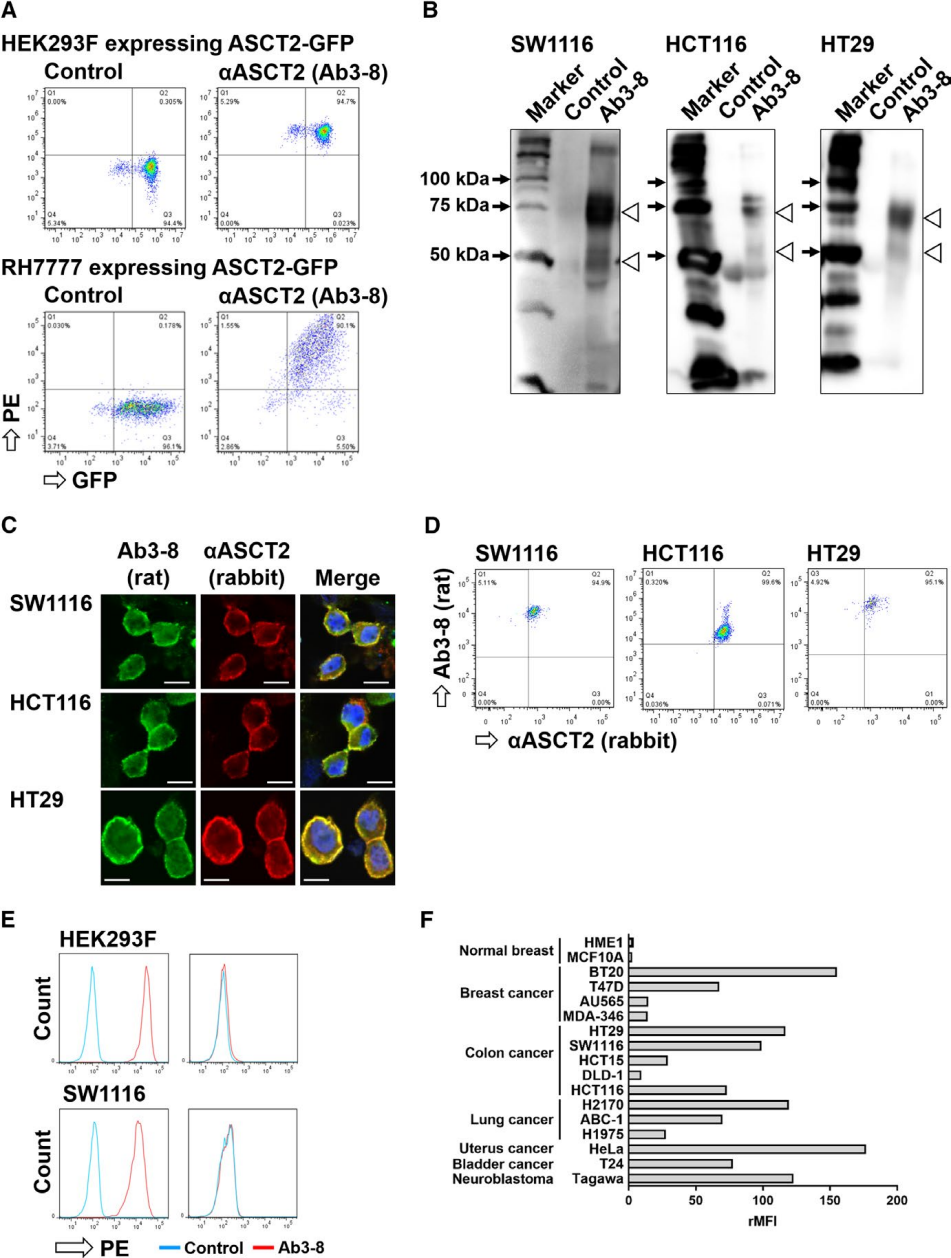
Specificity of the Ab3‐8 anti‐human ASCT2 rat mAb. A, FCM analysis of Ab3‐8 against HEK293 (upper) and RH7777 (lower) cells expressing ASCT2‐GFP. PE, phycoerythrin. B, Immunoprecipitation. Lysates of SW1116, HCT116 and HT29 cells were immunoprecipitated with Ab3‐8, and then detected using a commercial anti‐ASCT2 rabbit mAb. Arrowhead, position of human ASCT2 proteins. C, PFA‐fixed cells were stained with a combination of Ab3‐8 and anti‐ASCT2 rabbit mAb, followed by Alexa Fluor 488‐conjugated anti‐rat IgG and Alexa Fluor 647‐conjugated anti‐rabbit IgG. Nuclei were counterstained with 4′,6‐diamidino‐2‐phenylindole (DAPI). Images were taken at 60 × magnification. Scale bar, 10 μm. D, PFA‐fixed cells were stained with the same antibodies as described above. E, *ASCT2*‐KO HEK293 and SW1116 cells were reacted with Ab3‐8, followed by PE‐conjugated anti‐rat IgG. The reactivity of Ab3‐8 with these cells was analyzed by flow cytometry. F, FCM analysis of cell surface expression of ASCT2 proteins in various human cell lines. The indicated cells were stained with Ab3‐8, followed by PE‐conjugated anti‐rat IgG. From values of the mean fluorescence intensity (MFI), the ratio (+mAb/ −mAb) of MFI (rMFI) was calculated.

We have previously established a screening method for mAbs using flow cytometry based on the reactivity with transfectants stably expressing GFP‐tagged target protein.^30^, ^31^ We examined the reactivity of rat antibodies in supernatants of hybridoma cultures with cells expressing human ASCT2‐GFP. The Ab3‐8 (IgG2a/κ) rat mAb definitely reacted with HEK293 cells expressing human ASCT2‐GFP, and with RH7777 cells expressing ASCT2‐GFP in a GFP expression level‐dependent manner (Figure 1A). Furthermore, immunoprecipitation analysis revealed that Ab3‐8 immunoprecipitated glycosylated 75‐ and non‐glycosylated 50‐kDa proteins in extracts prepared from three human colon cancer cell lines (Figure 1B). As anti‐ASCT2 rabbit mAb (D7C12) is available for biochemical experiments, we evaluated the specificity of Ab3‐8 by comparing staining pattern of Ab3‐8 with that of D7C12. On two‐color immunostaining of human colon cancer cell lines (SW1116, HCT116, and HT29), the components stained by Ab3‐8 were merged with components stained by the anti‐ASCT2 rabbit mAb (Figure 1C). In FCM, the Ab3‐8‐positive populations in these colon cancer cells were also stained by the anti‐ASCT2 rabbit mAb (Figure 1D). Next, we examined the reactivity of Ab3‐8 with *ASCT2*‐KO cells using the CRISPR‐Cas9 system. Ab3‐8 did not react with *ASCT2*‐KO‐HEK293 and SW1116 cells (Figure 1E). Furthermore, Ab3‐8 reacted with various human cancer cells, but its reactivity with non‐cancer cells was weak (Figure 1F). Among these cell lines, SW1116, HCT116, DLD‐1, and HCT15 cells harbor a *KRAS* mutation, whereas other cell lines have wild‐type *KRAS*.^32^, ^33^, ^34^, ^35^, ^36^, ^37^

### 3.2 Functional activity of Ab3‐8

**Figure 2:**
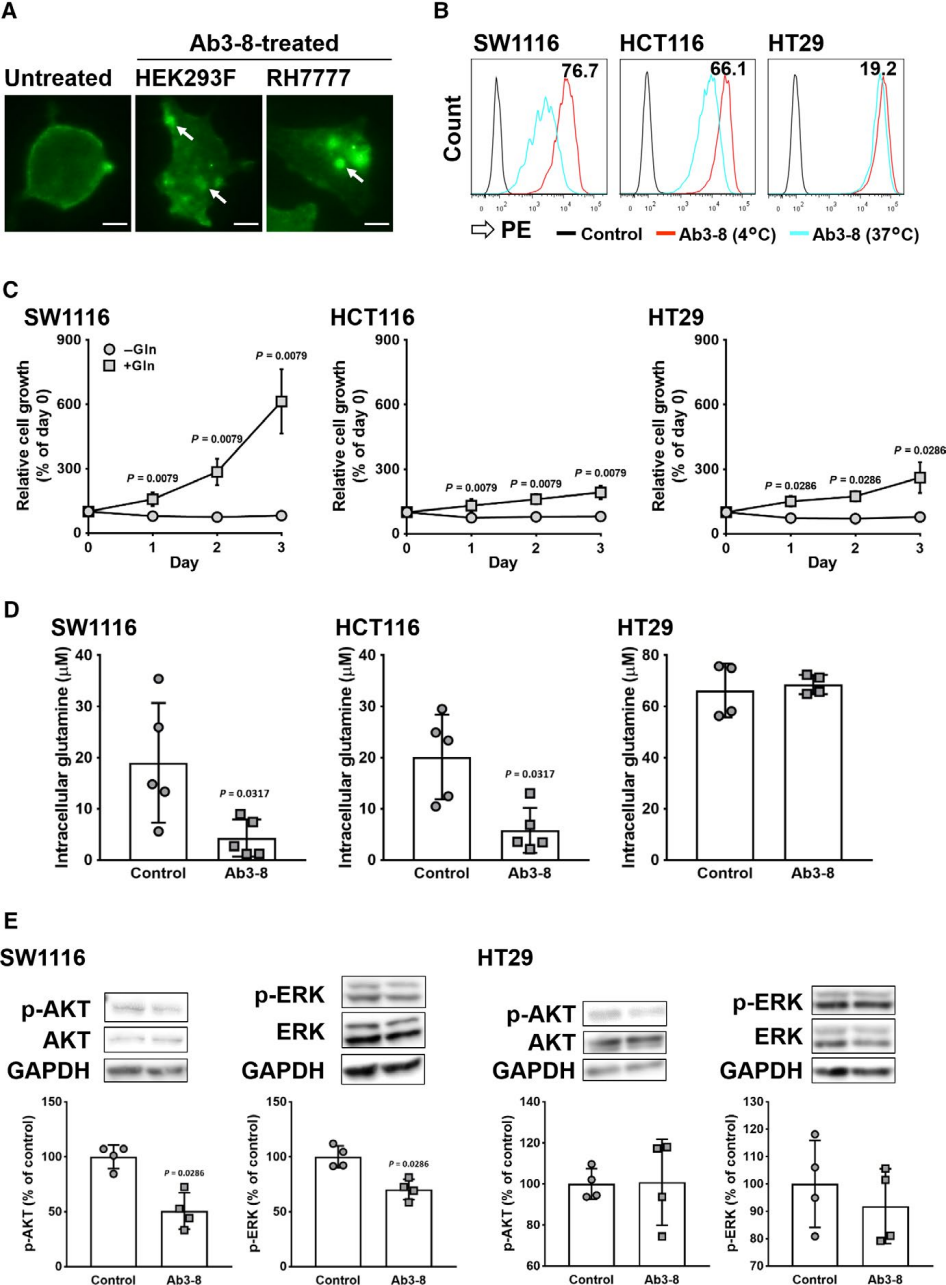
Inhibitory effects of Ab3‐8 on ASCT2 functions. A, Microscopy study of ASCT2 internalization. HEK293 and RH7777 cells overexpressing ASCT2‐GFP were reacted with Ab3‐8 at 37°C for 1 h. Images were taken at 20 × magnification. Arrows indicate internalized ASCT proteins. Scale bar, 10 μm. B, Quantitative analysis of ASCT2 internalization using FCM. Human CRC cells were incubated with Ab3‐8 at 4°C or 37°C for 1 h, followed by PE‐conjugated secondary antibody. Numeric data in each panel indicate internalization (%) calculated from the reactivity (MFI) of Ab3‐8 with CRC cells at 37°C and 4°C. C, Glutamine‐dependent cell growth. Cells were cultured with or without glutamine (2 mmol/L) for 3 d. Relative cell growth was measured every 24 h. Each dot represents the mean of four (HT29) or five (SW1116 and HCT116) independent experiments. D, Intracellular glutamine uptake. Cells were incubated in PBS in the presence of Ab3‐8 (30 μg/mL) for 2 h, and then treated with 2 mmol/L glutamine for 10 min. Each dot represents value of individual experiments. E,Cells were incubated in serum‐ and glutamine‐free media in the presence of Ab3‐8 for 24 h, and then treated with 2 mmol/L glutamine for 15 min. Total and phosphorylated (p‐) AKT and ERK protein levels were measured by western blotting. GAPDH was used as a loading control. Each dot represents value of individual experiments. Data are expressed as mean ± SD of at least four independent experiments

We investigated the functional activity of Ab3‐8. One of the anti‐cancer mechanisms mediated by mAb is internalization; that is, cell surface targets are transferred into the intracellular part after the binding of antibodies.^38^, ^39^, ^40^ Membrane localization of green fluorescence was observed in untreated HEK293 (Figure 2A, left) and RH7777 (data not shown) cells expressing ASCT2‐GFP, whereas intracellular localization of green fluorescence was observed in cells treated with Ab3‐8 (Figure 2A, middle and right). Furthermore, we carried out FCM analysis to quantitate cell surface expression of ASCT2 proteins. In SW1116 and HCT116 cells, which harbor *KRAS* mutation, treatment with Ab3‐8 at 37°C reduced surface expression of ASCT2 compared with at 4°C. On the other hand, treatment with Ab3‐8 had little effect on ASCT2 surface expression levels in HT29 with no *KRAS* mutation (Figure 2B).

Next, we examined whether growth of *KRAS*‐mutated and wild‐type *KRAS* colon cancer cells is dependent on glutamine (Figure 2C). All cell lines did not grow under glutamine‐depleted conditions. However, SW1116 cells markedly proliferated in the presence of glutamine. Although HCT116 and HT29 cells proliferated in the presence of glutamine, the proliferation rate of these cells was very small as compared with that of SW1116 cells.

Since ASCT2 is a major glutamine transporter in cancer cells,^18^ we examined whether Ab3‐8 inhibited intracellular glutamine transport. As shown in Figure 2D, Ab3‐8 markedly inhibited glutamine uptake into SW1116 and HCT116 cells. On the other hand, treatment with Ab3‐8 had no effect on intracellular glutamine concentration in HT29 cells.

AKT and ERK signaling pathways have been reported to be involved in growth and survival of cancer cells.^41^, ^42^, ^43^ As shown in Figure 2E, treatment with Ab3‐8 suppressed the phosphor‐ (p‐) AKT and p‐ERK levels in SW1116 cells. However, consistent with the glutamine uptake assay, Ab3‐8 did not change the p‐AKT and p‐ERK levels in HT29 cells.

### 3.3 Effects of Ab3‐8 on in vivo tumor growth of colon cancer cells

**Figure 3:**
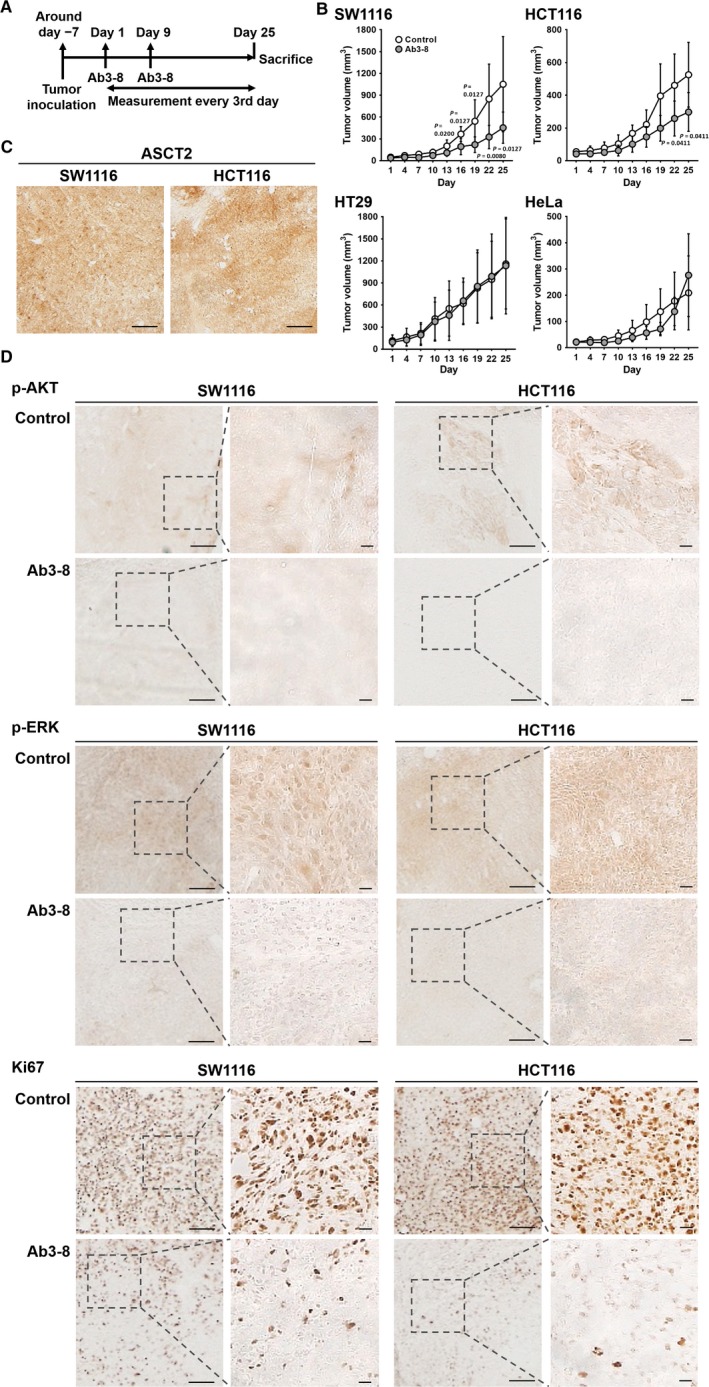
Anti‐tumor effects of Ab3‐8. Cells were injected subcutaneously into the left/right flanks of male nude mice. A, Time course of the in vivo anti‐tumor study. B, Tumor volumes of CRC and HeLa cells were measured every 3 d using digital calipers, and were quantified using the formula: volume [mm^3^] = (length [mm]) × (width [mm])^2^ × 0.5. Ab3‐8 (100 μg/mouse) was intraperitoneally administered on days 1 and 9. Each dot represents the mean of 4‐9 mice. Data are expressed as the mean ± SD. Tumor volume of individual mice shows in Figure S3. C,Tumor sections from control mice were subjected to immunoperoxidase staining using Ab3‐8. Scale bar, 20 μm. D, Tumor sections were subjected to immunoperoxidase staining using the indicated antibodies. Representative images of the SW1116‐ and HCT116‐derived tumor sections, which immune labeled with p‐AKT, p‐ERK and Ki67. Images were obtained at 4 × magnification. Scale bar, 20 μm. Insets, higher magnification of the boxes. Images were obtained at 20 × magnification. Scale bar, 20 μm

We have next evaluated whether Ab3‐8 inhibits tumor growth of *KRAS*‐mutated colon cancer cells (Figure 3A). Treatment with Ab3‐8 slowed tumor growth of both SW1116 and HCT116 (Figure 3B). Similar to the in vitro cultured colon cancer cells (Figure 1F), ASCT2 proteins were highly expressed in these in vivo colon cancer tumors (Figure 3C). In addition to inhibitory effects on tumor growth, Ab3‐8 suppressed the expression of p‐AKT, p‐ERK, and Ki67 proteins (Figure 3D). However, Ab3‐8 had no effect on the tumor growth of HT29 and HeLa cells, both of which do not have a *KRAS* mutation (Figure 3B).

## 4 DISCUSSION

*KRAS* mutations are observed in many human malignancies, such as non‐small‐cell lung cancer,^44^, ^45^ CRC,^3^, ^46^ and pancreatic ductal adenocarcinoma^47^ and are related to their oncogenesis. However, there are no effective methods to treat *KRAS*‐mutated cancers.

Cancer cells increase the demand for amino acids compared with normal cells because of their rapid metabolism and proliferation. To fulfill this demand, cancer cells upregulate the expression of amino acid transporters on the cell membrane.^11^ In particular, glutamine is one of the most important nutrients in cancer cells because of enhanced glutaminolysis.^11^, ^12^, ^13^ In this context, glutamine is estimated as a conditional essential amino acid. Previous studies have revealed that ASCT2 is a major glutamine transporter, and is upregulated in multiple cancer cell types such as breast, gastric, prostate, and CRC.^18^, ^48^ Furthermore, expression of ASCT2 is correlated with malignant features.^18^ Regarding CRC, several studies have suggested that glutamine metabolism plays an essential role in cellular growth.^49^ Pharmacological blockade and genetic silencing of *ASCT2* can suppress cancer cell proliferation and tumor growth through the inhibition of glutamine influx.^19^, ^20^, ^21^, ^22^, ^23^, ^24^, ^50^ Taken together, these findings suggest that ASCT2 is a promising target for cancer therapy.

Recently, production of anti‐human ASCT2 mAbs was reported, and these mAbs suppressed glutamine‐dependent cancer cell growth.^51^ However, it remains unclear whether these mAbs have in vivo anti‐tumor activity against *KRAS*‐mutated cancers. Here, we successfully established a novel hybridoma clone secreting the Ab3‐8 mAb against human ASCT2. Ab3‐8 reacted with transfectants expressing human ASCT2‐GFP in a GFP‐expression‐dependent manner. Furthermore, immunoprecipitation assay revealed that Ab3‐8 detected unglycosylated 50‐kDa and glycosylated mature 75‐kDa ASCT2 proteins in human colon cancer cells. These results indicate that Ab3‐8 specifically reacts with human ASCT2 proteins.

We attempted to directly observe Ab3‐8‐mediated internalization of ASCT2 proteins by means of a fluorescent microscopy. Treatment with Ab3‐8 resulted in increased green fluorescent dots in the intracellular region. The dots are assumed to be endocytic vesicles. However, it remains unclear whether Ab3‐8 is released into the cytoplasm or is degrade in the lysosome after the internalization. To confirm this, it needs to further stain lysosome using antibody against the lysosome marker or dye as other groups have done.^52^, ^53^

We found that Ab3‐8 inhibited the tumor growth of SW1116 and HCT116 colon cancer cells. Furthermore, treatment with Ab3‐8 reduced intracellular glutamine concentrations in both cell lines. On the other hand, Ab3‐8 had no effects on intracellular glutamine concentration and in vivo tumor growth in HT29 cells. We assume that cause of these results would be attributed to difference in Ab3‐8‐mediated internalization of ASCT2 proteins. FCM analysis revealed that treatment with Ab3‐8‐induced internalization of ASCT2 in both SW1116 and HCT116 cells, but not in HT29 cells. Ma et al^54^ had reported that treatment with L‐γ‐glutamyl‐p‐nitroanilide, an ASCT2 inhibitor, decreased glutamine consumptions in wild‐type *KRAS* Caco‐2 cells as well as in *KRAS*‐mutated SW480 cells. Taken together, Ab3‐8 does not directly inhibit function of ASCT2, but suppresses glutamine uptake through internalization of ASCT2 expressing on cell surface. Although Ab3‐8 binds to ASCT2 proteins expressed on membrane of HT29 cells, the reason why Ab3‐8 did not induce internalization in HT29 cells remains unclear.

HT29 cell line does not harbor *KRAS* mutation, whereas the cell line harbors *BRAF* and *PIK3CA*, the most commonly altered genes in human CRC, mutations.^32^ For this reason, we cannot accurately judge the effects of Ab3‐8 on tumor growth of *KRAS*‐mutated cancer. Therefore, we used HeLa cells because the cell line harbors wild‐type *KRAS*, *BRAF*, and *PIK3CA* genes.^35^, ^55^, ^56^ Similar to HT29 cells, treatment with Ab3‐8 did not affect tumor growth of HeLa cells, although we found that the expression of ASCT2 proteins in HT29 and HeLa cells was higher than that in SW1116 and HCT116 cells. Many studies have reported the importance of glutamine metabolism in *KRAS*‐mutated cancers.^15^, ^16^, ^17^ For this reason, glutamine is likely more important for the survival of SW1116 and HCT116 cells than for HT29 and HeLa cells. In fact, we found that SW1116 cells effectively grew under the glutamine‐enriched conditions as compared with HT29 cells. Accordingly, we speculate that inhibition of ASCT2 function is more effective for *KRAS*‐mutated cancer than for wild‐type *KRAS* cancer regardless of the ASCT2 expression level. However, we found unexpectedly that glutamine‐dependent growth rate of HCT116 cells was nearly identical to that of HT29 cells. It would be of great interest to elucidate why Ab3‐8 had no effect on *KRAS* wild‐type tumors, and this could lead to the development of novel drugs for *KRAS*‐mutated cancers.

ASCT2 is also expressed in normal mouse tissues such as lung, skeletal muscle, and large intestine.^57^ However, Ab3‐8 did not recognize mouse ASCT2 protein (data not shown); therefore, we speculated that Ab3‐8 specifically bound to the inoculated tumor expressing human ASCT2 proteins. It was recently reported that *Slc1a5* knockout mice exhibit normal growth, survival, and development of adaptive immune cells.^58^, ^59^ This suggests that ASCT2‐targeted cancer therapy will be well tolerated with few adverse effects.

Although the importance of glutamine in *KRAS*‐mutated cancers has been investigated, the exact effects of the *KRAS* mutation status on ASCT2 function are unclear. KRAS regulates the expression of amino acid transporters including ASCT2, and glutamine uptake in *KRAS*‐mutated lung cancer cells.^60^ Furthermore, ASCT2 was upregulated in human CRC cells expressing mutant *KRAS* as compared with cells expressing wild‐type *KRAS*.^61^ Analysis of human CRC samples revealed a significant positive correlation between the *KRAS* mutation status and ASCT2 protein expression, and an association between tumor depth and ASCT2 expression.^25^ These findings suggest that regulation of the expression and function of ASCT2 is closely related to the *KRAS* mutation status. Further studies are needed to clarify the precise relationship between *KRAS* mutation and ASCT2, particularly how KRAS regulates ASCT2 expression.

ASCT2 is often expressed together with L‐type amino acid transporter 1 (LAT1)/CD98hc (SLC7A5/SLC3A2), a heterodimeric antiporter that exchanges large neutral amino acids.^48^, ^62^, ^63^ Both transporters have been implicated in cancer growth and mTOR signaling in many studies.^63^, ^64^ ASCT2 was proposed to take up glutamine, which then acts as an exchange substrate to accumulate leucine via LAT1/CD98hc. We have recently reported the anti‐tumor effects of a mAb against LAT1, which preferentially transports many essential amino acids.^29^ As tumor growth highly depends on the nutritional condition, maintaining a low‐nutrient environment is likely to induce tumor regression. Taken together, simultaneous inhibition of ASCT2 and LAT1 may be more effective than their individual inhibition for treating cancer.

In conclusion, this study demonstrated that the Ab3‐8 anti‐ASCT2 mAb inhibits the tumor growth of colon cancer cells with *KRAS* mutation by inhibiting glutamine uptake. Together with the finding that the growth of *KRAS*‐mutated cancers, including pancreatic and lung cancers, is strongly dependent on glutamine metabolism,^16^, ^65^ this study suggests inhibition of glutamine uptake as a potential therapeutic target in *KRAS*‐mutated various human malignancies.

## CONFLICT OF INTEREST

The authors declare no potential conflicts of interest.

## AUTHORS’ CONTRIBUTIONS

YH contributed to the study design, carried out almost all experiments, analyzed and interpreted the data, compiled all the figures, and wrote the manuscript. YM, SY, AY, NH, and SU carried out parts of the experiments. KM performed cDNA cloning. TM conceived and supervised the study, participated in its design and coordination, and contributed to the data analysis and interpretation. All authors have read and approved the final version of the manuscript.
