# Supplementary figures and images for: Anti‐tumor effects of an antagonistic mAb against the ASCT2 amino acid transporter on KRAS‐mutated human colorectal cancer cells
Source: Cancer Med. 2019 Nov 10;9(1):302–12. doi: 10.1002/cam4.2689 (PMC6943164; doi:10.1002/cam4.2689)

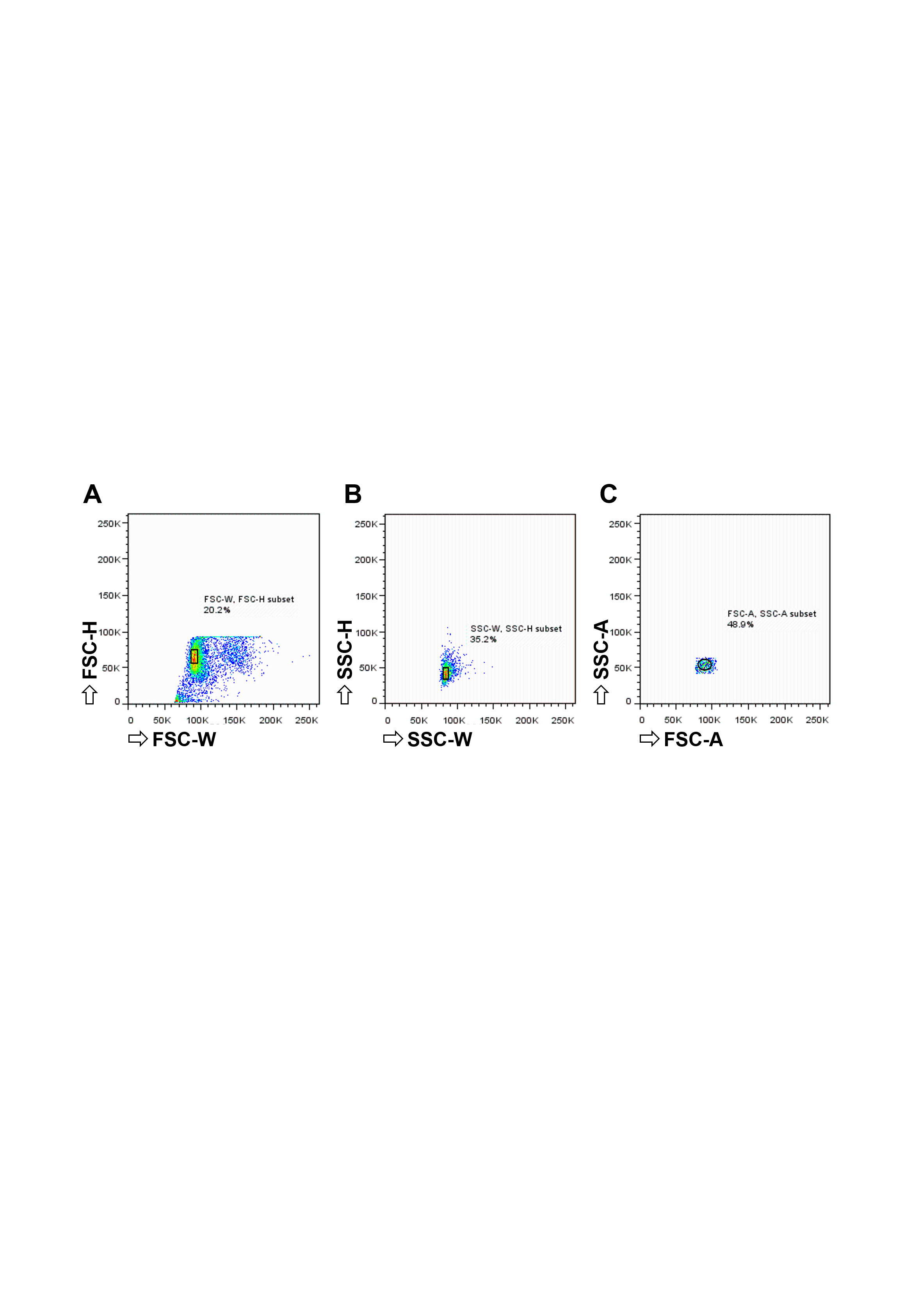

Supplement: Supplementary file 1 [file CAM4-9-302-s001.tif]

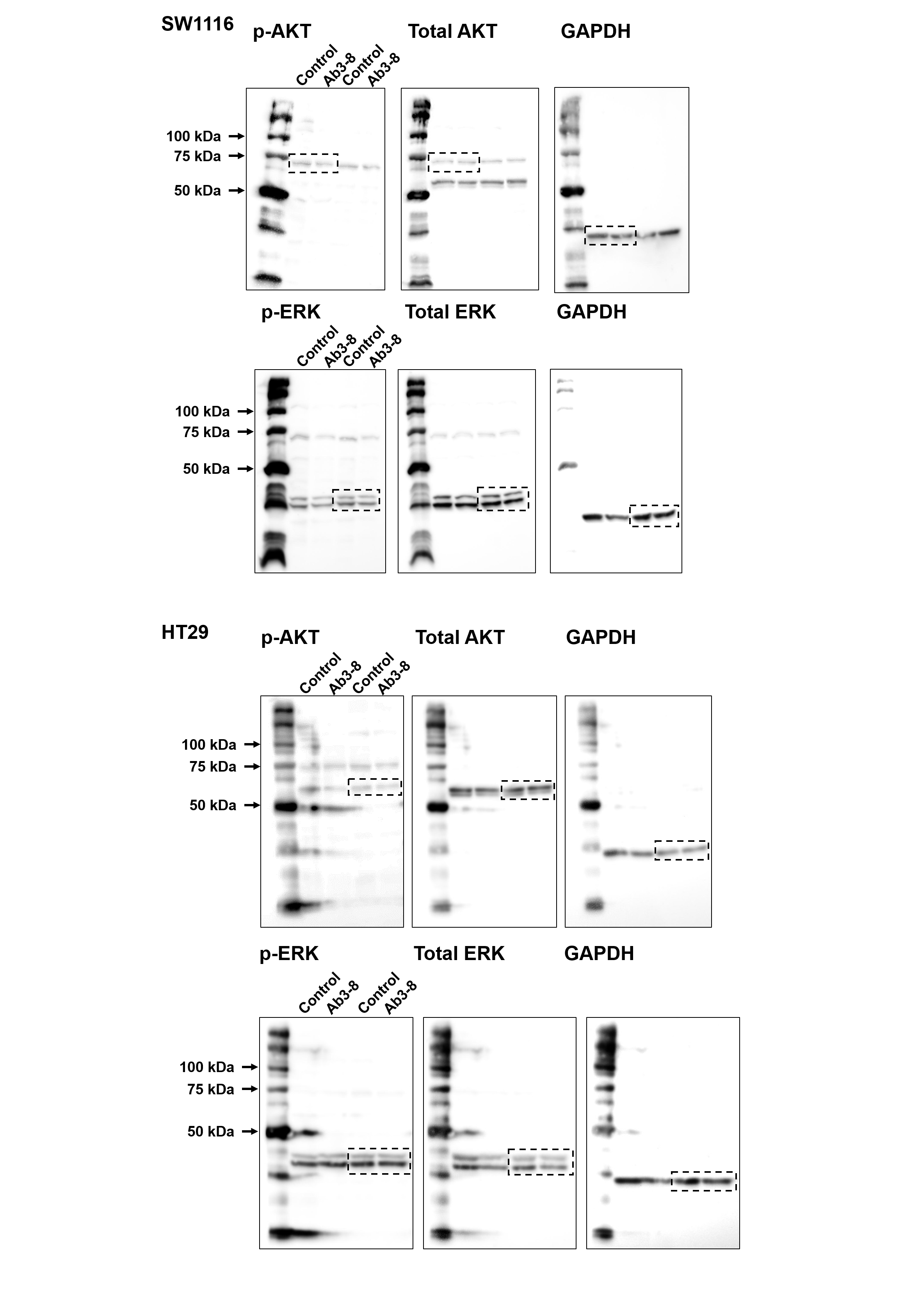

Supplement: Supplementary file 2 [file CAM4-9-302-s002.tif]

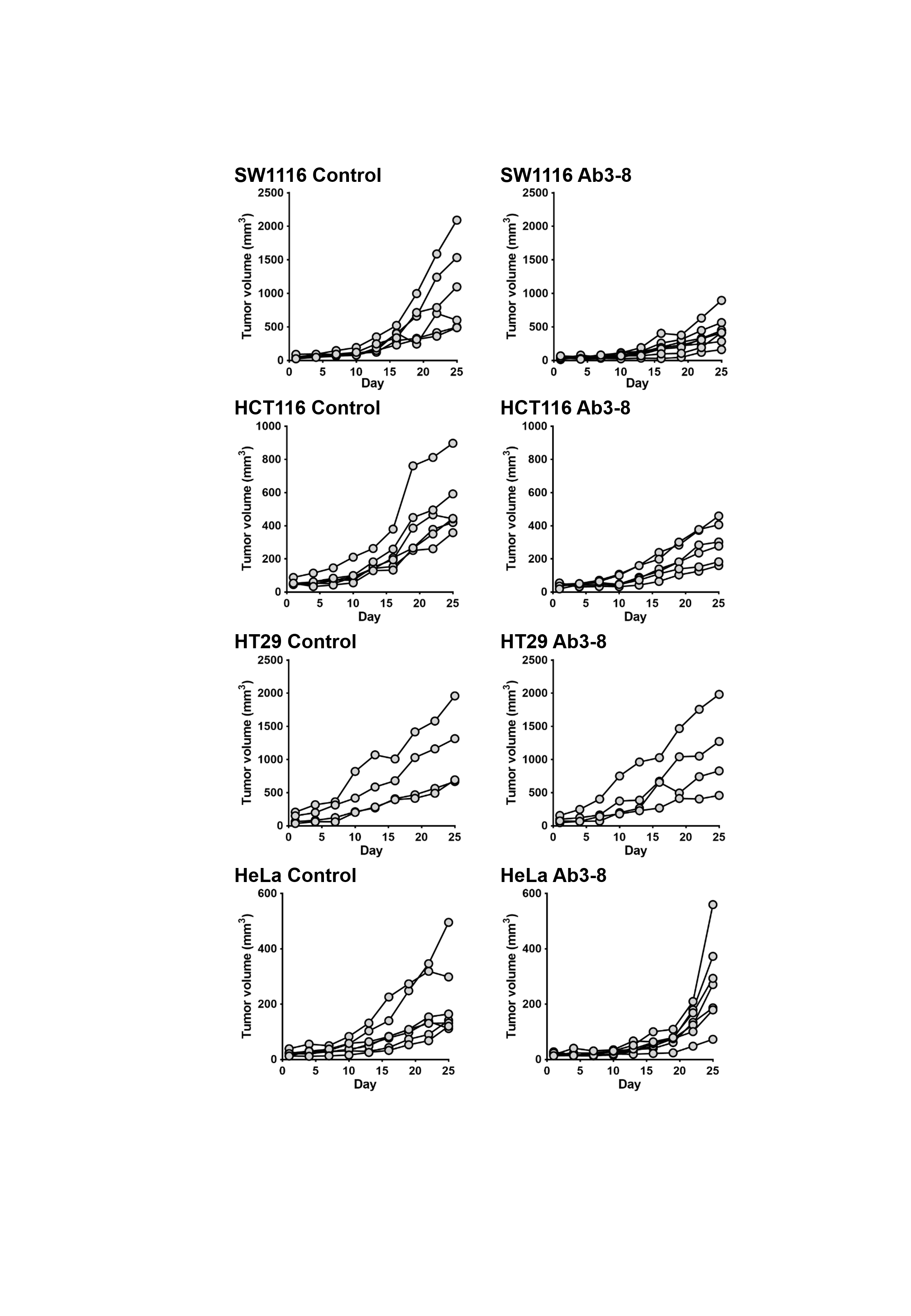

Supplement: Supplementary file 3 [file CAM4-9-302-s003.tif]
